# Supplementary material for: Oxytocin and vasopressin within the ventral and dorsal lateral septum modulate aggression in female rats
Source: Nat Commun. 2021 May 18;12:2900. doi: 10.1038/s41467-021-23064-5 (PMC8131389; doi:10.1038/s41467-021-23064-5)
Supplement: Supplementary file 3 — Description of Additional Supplementary Files [file 41467_2021_23064_MOESM3_ESM.docx]

**Description of Additional Supplementary Files**

File Name: **Supplementary Movie 1. Female aggression**

Description: Video depicting aggression in female Wistar rats.

File Name: **Supplementary Movie 2. Optogenetic stimulation of OXT axons**

Description: Video depicting the effect of stimulation of OXT axon on aggression in female Wistar rats.

File Name: **Supplementary Movie 3.** **Optogenetic stimulation of controls.**

Description: Video showing that blue-light does not affect the behavior of control female Wistar rats.
