# Supplementary material for: Oxytocin and vasopressin within the ventral and dorsal lateral septum modulate aggression in female rats
Source: Nat Commun. 2021 May 18;12:2900. doi: 10.1038/s41467-021-23064-5 (PMC8131389; doi:10.1038/s41467-021-23064-5)
Supplement: Supplementary file 8 — Reporting Summary [file 41467_2021_23064_MOESM8_ESM.pdf]

nature research

Corresponding author(s): Inga D. Neumann

Last updated by author(s): Feb 5, 2021

Reporting Summary

Nature Research wishes to improve the reproducibility of the work that we publish. This form provides structure for consistency and transparency in reporting. For further information on Nature Research policies, see our Editorial Policies and the Editorial Policy Checklist.

Statistics

For all statistical analyses, confirm that the following items are present in the figure legend, table legend, main text, or Methods section.

n/a	Confirmed	
☐	☒	The exact sample size (*n*) for each experimental group/condition, given as a discrete number and unit of measurement
☐	☒	A statement on whether measurements were taken from distinct samples or whether the same sample was measured repeatedly
☐	☒	The statistical test(s) used AND whether they are one- or two-sided<br>*Only common tests should be described solely by name; describe more complex techniques in the Methods section.*
☐	☒	A description of all covariates tested
☐	☒	A description of any assumptions or corrections, such as tests of normality and adjustment for multiple comparisons
☐	☒	A full description of the statistical parameters including central tendency (e.g. means) or other basic estimates (e.g. regression coefficient) AND variation (e.g. standard deviation) or associated estimates of uncertainty (e.g. confidence intervals)
☐	☒	For null hypothesis testing, the test statistic (e.g. *F*, *t*, *r*) with confidence intervals, effect sizes, degrees of freedom and *P* value noted<br>*Give P values as exact values whenever suitable.*
☒	☐	For Bayesian analysis, information on the choice of priors and Markov chain Monte Carlo settings
☒	☐	For hierarchical and complex designs, identification of the appropriate level for tests and full reporting of outcomes
☐	☒	Estimates of effect sizes (e.g. Cohen's *d*, Pearson's *r*), indicating how they were calculated

Our web collection on statistics for biologists contains articles on many of the points above.

Software and code

Policy information about availability of computer code

Data collection	All softwares used are mentioned in the methods session pages 14-21 :JWatcher (behavioral scoring); Heka (electrophysiological recordings); the Leica Application Suite X (immunohistochemistry image acquisition)
Data analysis	All the softwares used for data analysis are mentioned in the methods session pages 14-21 Fiji ImageJ (December 2007), Origin Lab (2019); Graphpad Prism 6.0;

For manuscripts utilizing custom algorithms or software that are central to the research but not yet described in published literature, software must be made available to editors and reviewers. We strongly encourage code deposition in a community repository (e.g. GitHub). See the Nature Research guidelines for submitting code & software for further information.

Data

Policy information about availability of data

All manuscripts must include a data availability statement. This statement should provide the following information, where applicable:

- Accession codes, unique identifiers, or web links for publicly available datasets
- A list of figures that have associated raw data
- A description of any restrictions on data availability

The data that support the findings of this study are available from the corresponding author upon reasonable request.

Field-specific reporting

Please select the one below that is the best fit for your research. If you are not sure, read the appropriate sections before making your selection.

- [x] Life sciences
- [ ] Behavioural & social sciences
- [ ] Ecological, evolutionary & environmental sciences

For a reference copy of the document with all sections, see nature.com/documents/nr-reporting-summary-flat.pdf

Life sciences study design

All studies must disclose on these points even when the disclosure is negative.

Sample size	Sample size was determined to be adequate based on the magnitude and consistency of measurable differences between groups and/or experimental conditions. Group sizes were estimated (according to "G*POWER" and 2-sided non-parametric test; ANOVA and posthoc test, or U-test according to Wilcoxon-Mann-Whitney) .Biological variability corresponding to behavioral variability is between 20-25% was also taken in consideration when determining the group sizes.
Data exclusions	Only anatomical outliers in stereotaxic cannula implantation or animals who did not show viral infection were excluded from analyses. For the electrophysiological experiments, leaky cells with a holding current beyond ~ -30 pA were not further used for experimentation, meaning that they did not receive pharmacological treatment. See methods page 1-20
Replication	All experiments included in the manuscript were performed in different cohorts. The data in figure 1, for example, was performed in three different cohorts demonstrating replication and homogenous effects of isolation and training although the intensity of aggression differed between cohorts. Moreover, all cohorts tested for pharmacological experiments showed homogenous effects of the drug although the baseline levels of aggression differed from one cohort to the other. I.c.v. experiments were performed in 2 cohorts of animals (except AVP experiment which was performed in 3 cohorts). Local infusions and MD were performed in 2 different cohorts except GH dLS V1aR antagonist infusion which was performed only once. Opto and chemogenetics experiments were performed in a single cohort. Again we reinforce here that our isolation plus training protocol was able to enhance aggression in all the cohorts evaluated in this manuscript although the absolute levels of aggression differed depending whether the animals had surgery or not for more information please see methods pages 1-20 and 1st rebuttal letter answer to reviewer #1.
Randomization	For pharmacological experiments in highly aggressive females, rats were divided into the respective VEH or treatment group based on their average aggression data from FIT 3. Group-housed animals were randomized in different groups. All animals used in one group came from different litters in order to increase the diversity within a group. See methods 1-20
Blinding	Blinded data collections was done when possible. However, as V.E.M.O. conducted most of the experiments on his own blinded data collection for pharmacological, as well as MD and neural activity experiments was not possible. However all the data was analyzed by an observer blinded with respect to groups and treatments. See methods 1-20

Reporting for specific materials, systems and methods

We require information from authors about some types of materials, experimental systems and methods used in many studies. Here, indicate whether each material, system or method listed is relevant to your study. If you are not sure if a list item applies to your research, read the appropriate section before selecting a response.

Materials & experimental systems

n/a	Involved in the study	
☐	☒	Antibodies
☒	☐	Eukaryotic cell lines
☒	☐	Palaeontology and archaeology
☐	☒	Animals and other organisms
☒	☐	Human research participants
☒	☐	Clinical data
☒	☐	Dual use research of concern

Methods

n/a	Involved in the study	
☒	☐	ChIP-seq
☒	☐	Flow cytometry
☒	☐	MRI-based neuroimaging

Antibodies

Antibodies used	All the antibodies used can be found in supplementary table 5
Validation	Information on validation of anti-oxytocin (PS38, mouse) antibody is reported in previous publications from our lab, please see methods and Dr. Harold Gainer (NIH, Bethesda) papers. All other antibodies validations are available on the description files of the respective companies websites. For more information please check our references in the method section. Specifically, anti-pERK antibody and streptavidin staining have been previously used and validated in our lab, please see methods and references. Anti-ERa and Anti-Somatostatin antibodies were validated by the respective companies in cell culture for IHC. Moreover, without primary and without secondary controls were ran in parallel to the experimental animals to check for antibody specificity.

Animals and other organisms

Policy information about studies involving animals; ARRIVE guidelines recommended for reporting animal research

Laboratory animals	As described in the methods 10-14 weeks old female Wistar rats were used for behavioral experiments whereas juvenile (p20-25) female Wistar rats were used for electrophysiology experiments.
Wild animals	N.A. No wild animals were used on this study.
Field-collected samples	N.A.
Ethics oversight	All procedures were conducted following the Guidelines for the Care and Use of Laboratory Animals of the Local Government of Oberpfalz and Unterfranken. Animal Liscence number 54.2532.1-24/13

Note that full information on the approval of the study protocol must also be provided in the manuscript.
